# Role of MiR-325-3p in the Regulation of CFL2 and Myogenic Differentiation of C2C12 Myoblasts

**DOI:** 10.3390/cells10102725

**Published:** 2021-10-12

**Authors:** Mai Thi Nguyen, Wan Lee

**Affiliations:** 1 Department of Biochemistry, Dongguk University College of Medicine, 123 Dongdae-ro, Gyeongju 38066, Korea; nguyenmainhp@gmail.com; 2 Channelopathy Research Center, Dongguk University College of Medicine, 32 Dongguk-ro, Goyang 10326, Korea

**Keywords:** microRNA, miR-325-3p, CFL2, palmitic acid, proliferation, differentiation

## Abstract

Skeletal myogenesis is required to maintain muscle mass and integrity, and impaired myogenesis is causally linked to the etiology of muscle wasting. Recently, it was shown that excessive uptake of saturated fatty acids (SFA) plays a significant role in the pathogenesis of muscle wasting. Although microRNA (miRNA) is implicated in the regulation of myogenesis, the molecular mechanism whereby SFA-induced miRNAs impair myogenic differentiation remains largely unknown. Here, we investigated the regulatory roles of miR-325-3p on CFL2 expression and myogenic differentiation in C2C12 myoblasts. PA impeded myogenic differentiation, concomitantly suppressed CFL2 and induced miR-325-3p. Dual-luciferase analysis revealed that miR-325-3p directly targets the 3′UTR of *CFL2*, thereby suppressing the expression of CFL2, a crucial factor for actin dynamics. Transfection with miR-325-3p mimic resulted in the accumulation of actin filaments (F-actin) and nuclear Yes-associated protein (YAP) in myoblasts and promoted myoblast proliferation and cell cycle progression. Consequently, miR-325-3p mimic significantly attenuated the expressions of myogenic factors and thereby impaired the myogenic differentiation of myoblasts. The roles of miR-325-3p on CFL2 expression, F-actin modulation, and myogenic differentiation suggest a novel miRNA-mediated regulatory mechanism of myogenesis and PA-inducible miR-325-3p may be a critical mediator between obesity and muscle wasting.

## 1. Introduction

Skeletal muscle is essential for proper physical and systemic homeostasis, including locomotion, metabolism, and respiration [1]. The maintenance of skeletal muscle mass and integrity is regulated by myogenesis, which involves satellite cell activation, myoblast differentiation, and myotube formation [2]. Accordingly, dysregulation of myogenesis induces muscle wasting diseases, including sarcopenia and cachexia, and increases the risks of frailty, morbidity, and mortality [2,3]. Numerous studies have indicated that muscle wasting is derived from diverse conditions that impede myogenesis, such as oxidative stress, mitochondrial dysfunction, and senescence [3,4]. Recently, it was suggested that obesity exacerbates the detrimental consequences of muscle wasting and leads to sarcopenic obesity [5,6]. Excess uptake of saturated fatty acids (SFA) increases intramuscular fat infiltration and frequently provokes muscle wasting [7,8]. Furthermore, many studies have shown that certain microRNAs (miRNAs) dysregulated by SFA, and obesity are associated with muscle wasting [9,10].

MiRNAs are a class of short non-coding RNAs and play essential roles in a broad range of biological processes by targeting the 3′UTR regions of target mRNAs [11,12]. For this reason, aberrant miRNA expression is intimately associated with the pathogeneses of numerous diseases, including cancer, cardiovascular diseases, and metabolic disorders [13]. Recently, many studies have focused on the regulatory roles of miRNAs in muscle homeostasis, muscle wasting, and other myopathies [14,15]. Accumulating evidence indicates that several miRNAs are involved in muscle wasting through their inhibitory effects on myogenesis [9,16]. Nevertheless, the molecular mechanism whereby SFA-induced miRNAs suppress myogenic differentiation remains largely unknown.

Actin remodeling, coordinated by actin-binding proteins, modulates the cytoskeletal dynamics necessary for myoblast proliferation and differentiation [17,18]. Cofilin 2 (CFL2) is a skeletal muscle-specific actin-binding protein and belongs to the actin-depolymerizing factor (ADF)/cofilin family [19,20]. CFL2 plays an essential role in actin remodeling by severing or depolymerizing filamentous actin (F-actin), which is involved in muscle development and maintenance [19,20]. In a mouse model, the functional ablation of CFL2 was associated with skeletal muscle wasting accompanied by F-actin accumulation [21]. In addition, CFL2 knockout disrupted sarcomere structure and integrity with enhanced actin polymerization [22]. Furthermore, CFL1-mediated actin remodeling has been shown to regulate cell proliferation associated with myogenic differentiation [23,24]. In a previous study, we found that CFL2 knockdown by siRNA promoted myoblast proliferation and consequently inhibited myogenic differentiation in C2C12 cells [25]. Although CFL2 is known to be crucial for skeletal myogenesis and maintenance, its regulation by miRNAs during myogenic differentiation has not been explored.

Here, we investigated the role of SFA-induced miRNA on myogenic differentiation. We found that miR-325-3p, markedly induced by palmitic acid (PA) in myoblasts, regulates CFL2 expression directly. We also showed that miR-325-3p plays a crucial role in cell proliferation, myogenic factors expressions, and differentiation in myoblasts. Our findings regarding the regulatory functions of miR-325-3p on myogenesis increase understanding of the mechanism of muscle wasting in the background of obesity and will provide a novel diagnostic and therapeutic target for muscle wasting and sarcopenic obesity.

## 2. Materials and Methods

### 2.1. Cell Culture, Differentiation and PA Treatment

C2C12 myoblasts, an immortalized murine muscle progenitor cell line (ATCC), were maintained in a growth medium (GM; Dulbecco’s modified Eagle’s medium (DMEM) containing 10% fetal bovine serum and 1% penicillin/streptomycin) (Gibco, Carlsbad, CA, USA) at 37 °C in a 5% CO_2_ humidified incubator. For the biochemical study, cells were seeded on 6-well plates (Thermo Fisher Scientific, Waltham, MA, USA) at a density of 1.3 × 10^5^ cells/well in 2 mL of GM. After 24 h, cells were transiently transfected with indicated oligonucleotides using Lipofectamine 2000 (Invitrogen, Waltham, MA, USA) according to the manufacturer’s instructions. When cells reached 80–90% confluence, myoblasts were differentiated to myotubes by switching to a differentiation medium (DM; DMEM containing 2% dialyzed horse serum and 1% penicillin/streptomycin). When necessary, cells were treated with BSA-conjugated PA (100 μM) for 24 h in GM before differentiation as described previously [26]. Unless otherwise stated, all reagents and materials were purchased from Sigma-Aldrich (St. Louis, MO, USA).

### 2.2. Transfection of Oligonucleotides

C2C12 myoblasts were transfected with scrambled control RNA (scRNA), CFL2 siRNA (siCFL2), miR-325-3p mimic, or antimiR-325-3p (an inhibitor of miR-325-3p; a 2′-O-methyl-modified antisense oligonucleotide against mature miR-325-3p) from Genolution (Seoul, Korea) at final concentrations of 200 nM in a GM using Lipofectamine 2000. The sequences of the oligonucleotides used for this study are listed in Table S1.

### 2.3. RNA Extraction, PCR and Quantitative Real-Time PCR (qRT-PCR)

Total RNA of C2C12 cells was extracted using a Qiazol reagent and miRNeasy Mini Kit (Qiagen, Hilden, Germany) 24 h after transfection. The quality and concentration of RNAs were assessed by gel electrophoresis and a UV-1700 PharmaSpec spectrophotometer (Shimadzu, Kyoto, Japan). The RNA was then reverse-transcribed using the miScript II RT Kit (Qiagen). To determine mRNA and miRNA expression levels, *q*RT-PCR and RT-PCR were conducted using specified primers, SYBR Green I, and iTaq polymerase (Promega, Medison, WI, USA) in conjunction with a Light-Cycler 480 (Roche Applied Science, Penzberg, Germany). Details of the primers used for RT-PCR and *q*RT-PCR and reaction conditions are described in Table S2. The 2^−ΔΔCt^ method was used to calculate relative mRNA expressions, and results were normalized by U6 snRNA expression.

### 2.4. Dual-Luciferase Assay

A segment of the murine CFL2 3′UTR (358-nt long) containing the potential miR-325-3p binding site (CFL2*wt*) was chemically synthesized by RT-PCR using specific primers listed in Table S2. The wild-type reporter construct was produced by subcloning CFL2*wt* into the pmirGLO vector (Promega) using *Sac*I and *Xba*I sites. Mutation of the miR-325-3p-binding site (CFL2*mut*) was synthesized by PCR-based site-directed mutagenesis using overlapping oligonucleotides (Table S2). For dual-luciferase target validation assays, C2C12 cells were plated in a 12-well plate at a density of 1 × 10^5^ cells/well. After 24 h, a pmirGLO vector containing CFL2*wt* or CFL2*mut* was co-transfected with scRNA or miR-325-3p mimic into cells using Lipofectamine 2000. The Dual-Luciferase Reporter Assay System 100 Kit (Abcam, Cambridge, UK) was used to determine luciferase activities 24 h after transfection as described recently [27].

### 2.5. Immunoblot Analysis

For protein preparation, C2C12 cells were collected by centrifugation and lysed using PBS containing 2% Triton X-100 and 1% phosphatase inhibitor cocktail II (Abcam) as previously described [28]. The NE-PER Nuclear and Cytoplasmic Extraction Reagents (Thermo Fisher Scientific) were used for nuclear and cytoplasmic protein fractionation. Protein concentrations were determined using the Bradford method, and then cell lysates were mixed with 2X Laemmli buffer and boiled for 10 min at 100 °C. Proteins were separated by SDS-PAGE at 20 µg of protein/lane and transferred to nitrocellulose membranes (Amersham, Germany). The membranes were then blocked with 5% skim milk in TTBS (0.5% TBS-Tween 20 in PBS) for 1 h and incubated with specific primary antibodies at 4 °C overnight (Table S3). The membranes were washed with TTBS 5 times and incubated with a secondary antibody for 1 h. Finally, protein bands were visualized using a Femto reagent (Thermo Fisher Scientific) with Fusion Solo (Vilber, Marne-la-Vallée, France), and their densities were determined by Evolution-Capt software (Vilber, Marne-la-Vallée, France). β-Actin protein levels were used for normalization.

### 2.6. Immunofluorescence Analysis

After transfection with the indicated oligonucleotides (Table S1), C2C12 cells were allowed to differentiate for five days. C2C12 myotubes were fixed and permeabilized using 4% paraformaldehyde and 0.3% Triton X-100 in PBS. Cells were then blocked with 3% BSA for 2 h at room temperature, incubated overnight with primary antibody (MyHC; 1:100 dilution), washed 3 times with PBS, and then incubated for an additional 1.5 h with secondary antibody (Alexa 488, Invitrogen; 1:200 dilution). In the case of F-actin staining, cells were incubated with 50 μg/mL FITC-conjugated phalloidin (P5282, Sigma, USA) for 40 min instead of MyHC antibody. Nuclei were counterstained with Hoechst 33342 (Invitrogen). All images were obtained using a fluorescence microscope (Leica Microsystems, Mannheim, Germany). Differentiation and fusion index were calculated as indicators of myotube formation. The differentiation index was defined as the number of nuclei expressed in MyHC-positive myotubes as a percentage of total nuclei. The fusion index was defined as the ratio of the total nuclei residing in myotubes with three or more nuclei. Myotube widths and MyHC-positive areas were analyzed using ImageJ software (ver 1.53, USA National Institutes of health, Bethesda, MA, USA).

### 2.7. Cell Proliferation Assays

EdU assays were applied to evaluate cell proliferation using the Click-iT EdU Cell Proliferation Kit (Invitrogen). Cells were plated in chamber slides at a density of 3 × 10^4^ cells/well. After 24 h of transfection, cells were treated for 4 h with EdU (10 μM), fixed with formaldehyde (4%) for 10 min, and permeabilized with Triton X-100 (0.3%) in PBS for 15 min. Then, cells were incubated with 0.3 mL of Click-iT reaction cocktail for 20 min, and nuclei were labeled for an additional 15 min with Hoechst 33342. Each image was taken with a Leica microscope. The total number of cells and the number of EdU-positive cells were counted in random pictures, and ImageJ software was used to calculate the percentages of EdU-positive cells and the total number of nuclei. All experiments were conducted at least three times using at least five randomly selected fields/experiment.

### 2.8. Flow Cytometry

C2C12 cells were collected 24 h after transfection, resuspended at a density of 1 × 10^6^ cells, and centrifuged at 3000 rpm for 5 min at 4 °C. Pellets were washed three times with PBS, fixed with 70% ethanol overnight at 4 °C, and treated with 500 μL of Cell Cycle Kit solution (C03551, Beckman Coulter, Brea, CA, USA) for 20 min in the dark. Finally, cell cycle analyses were performed using a CytoFLEX (Beckman Coulter, Brea, CA, USA).

### 2.9. Database and Statistical Analysis

Using publicly accessible bioinformatics software (TargetScan: www.targetscan.org, Pictar: pictar.mdc-berlin.de), putative miR-325-3p binding sites on the 3UTR of *CFL2* mRNA were identified. Results are presented as the means ± standard errors of at least three independent experiments. The statistical analysis was performed using the Student’s *t*-test for unpaired data.

## 3. Results

### 3.1. PA Inhibited Myogenic Differentiation but Elevated miR-325-3p Expression

**Figure 1:**
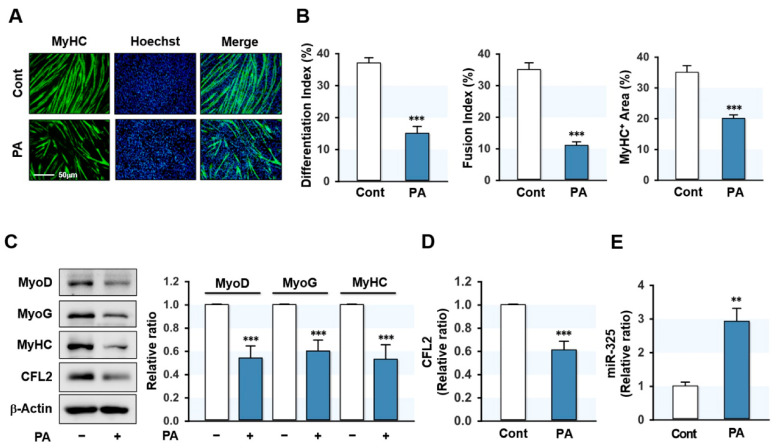
PA inhibited myoblast differentiation but enhanced miR-325-3p expression. (**A**) C2C12 myoblasts were pretreated with BSA-vehicle (Cont) or PA (100 μM) for 24 h and induced to differentiate for five days. Cells were subjected to immunocytochemistry with MyHC antibody (green) and Hoechst 33342 (blue) to verify differentiation. Scale bar: 50 μm. (**B**) Quantitative analysis of differentiation index, fusion index, and MyHC-positive area. (**C**,**D**) After pretreatment with PA, cells were differentiated for three days and immunoblotted with antibodies for myogenic factors (MyoD, MyoG, and MyHC) and CFL2. Intensities were normalized versus β-actin. (**E**) The expressions of miR-325-3p were determined by *q*RT-PCR and normalized versus U6. Immunoblot and *q*RT-PCR results are shown as relative ratios versus control. All results are presented as the means ± SEMs (*n* > 3), and levels of significance are presented as **, *p* < 0.01; ***, *p* < 0.001 vs. controls.

Since CFL2 is necessary for myoblast differentiation [25], we investigated how CFL2 expression during differentiation is affected by SFA in myoblasts. C2C12 cells were treated with PA (100 μM), the most abundant dietary SFA, for 24 h and then differentiated up to five days. Myoblast differentiation was then evaluated according to the myogenic factors expressions and myotube formation. PA-treatment remarkably decreased the MyHC-positive area and suppressed myoblast differentiation and fusion in C2C12 cells as determined by immunocytochemistry and quantitative image analysis (Figure 1A,B). In agreement with immunocytochemistry findings, PA significantly suppressed the levels of MyoD, MyoG, and MyHC (Figure 1C), indicating that PA dramatically impeded myogenic factors expressions and differentiation in C2C12 myoblasts. Interestingly, under these conditions, the expression of CFL2 was significantly diminished by PA (Figure 1C,D). These results suggest that impaired myogenic differentiation by PA is associated with CFL2 suppression in myoblasts. Next, we investigated whether specific miRNAs upregulated by PA are implicated in CFL2 suppression in myoblasts. According to microarray results, the expression of miR-325-3p, which was predicted to target 3′UTR of *CFL2* with a high probability according to the miRNA target analysis using TargetScan and miRWalk, was upregulated >1.5-fold in PA-treated myoblasts (Supplementary Data). Therefore, miR-325-3p was chosen for further investigation because it has been supposed to be associated with muscle atrophy and dystrophy [29,30]. The *q*RT-PCR confirmed that PA raised miR-325-3p expression in myoblasts by 3-fold (Figure 1E). Collectively, PA was found to impair myogenic differentiation and suppress CFL2 expression but induce miR-325-3p expression in myoblasts.

### 3.2. MiR-325-3p Directly Targeted CFL2 3′UTR

**Figure 2:**
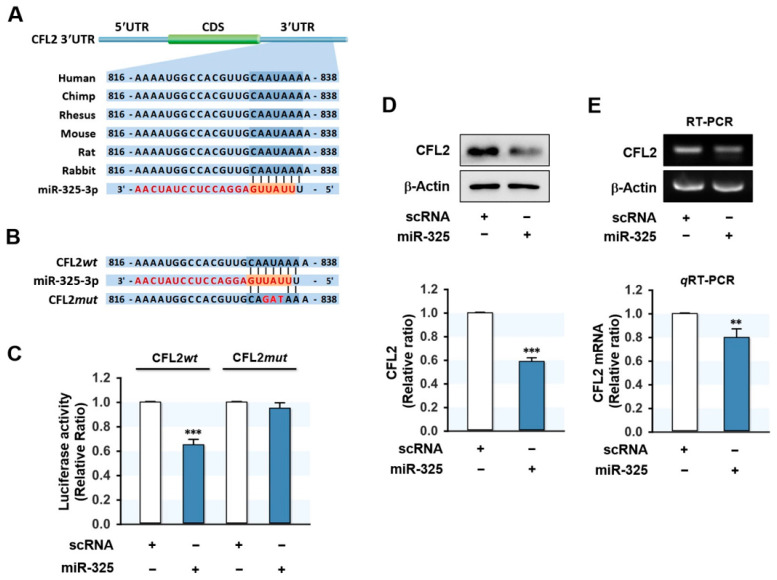
MiR-325-3p regulated CFL2 expression by binding to the 3′UTR of *CFL2*. (**A**) Putative binding sites of miR-325-3p on the 3′UTR fragments of *CFL2* mRNA. (**B**) Sequence alignment of miR-325-3p binding site with wild-type (CFL2*wt*) or mutant (CFL2*mut*) 3′UTR of *CFL2*. (**C**) MiR-325-3p mimic or scrambled control RNA (scRNA) were co-transfected with a dual-luciferase reporter construct containing CFL2*wt* or CFL2*mut* in C2C12 cells, and relative luciferase activity was measured 24 h after transfection. (**D**) CFL2 protein levels were analyzed 24 h after transfection with 200 nM of scRNA control or miR-325-3p mimic by immunoblotting. (**E**) The mRNA expressions were determined by RT-PCR (upper panel) and *q*RT-PCR (lower panel). Immunoblot and *q*RT-PCR results are shown as relative ratios versus scRNA control. All results are presented as the means ± SEMs (*n* > 3), and levels of significance are presented as **, *p* < 0.01; ***, *p* < 0.001 vs. scRNA controls.

Since miR-325-3p and CFL2 levels appeared to be inversely related in myoblasts, we next examined whether miR-325-3p directly targets and downregulates CFL2 expression. In silico analysis using TargetScan suggested that the 3′UTR of *CFL2* mRNA possesses a tentative binding site for the miR-325-3p seed sequence (Figure 2A). To investigate direct interaction between miR-325-3p and the *CFL2* 3′UTR, we constructed a luciferase reporter pmirGLO vector containing a *CFL2* 3′UTR segment of wild-type (CFL2*wt*) or mutant binding site (CFL2*mut*) for miR-325-3p (Figure 2B), and then co-transfected with miR-325-3p mimic or scRNA into C2C12 cells. As shown in Figure 2C, transfection of miR-325-3p mimic effectively reduced the luciferase activity of the wild-type (CFL2*wt*), whereas a mutant construct in the miR-325-3p binding site (CFL2*mut*) completely abolished the effect of miR-325-3p mimic on the luciferase activity of CFL2*wt*. Since direct binding between miR-325-3p and the 3′UTR of *CFL2* was confirmed by luciferase reporter analysis, we considered miR-325-3p induction might inhibit the protein level of CFL2 in myoblasts. To investigate this, we transfected C2C12 cells with scRNA or miR-325-3p mimic and then analyzed CFL2 protein and mRNA expressions. Transfection of miR-325-3p mimic decreased CFL2 protein significantly compared with scRNA transfection (Figure 2D). In addition, *CFL2* mRNA level was also decreased by miR-325-3p mimic as determined by RT-PCR and *q*RT-PCR (Figure 2E), indicating that miR-325-3p downregulated CFL2 expression by directly binding to the 3′UTR of *CFL2*.

### 3.3. MiR-325-3p Increased F-Actin and Nuclear Yes-Associated Protein (YAP)

**Figure 3:**
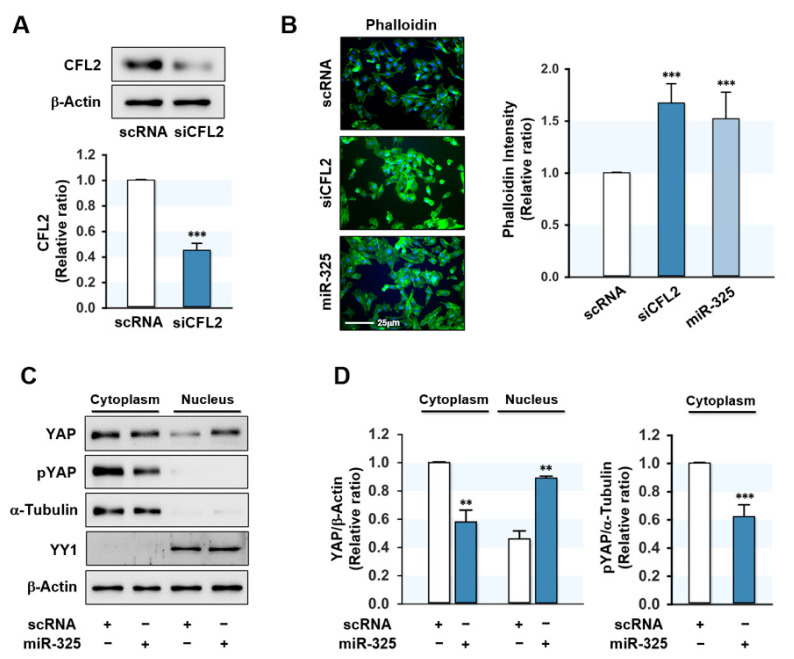
MiR-325-3p increased F-actin and nuclear YAP levels. (**A**) C2C12 myoblasts were transfected with 200 nM of scRNA or CFL2 siRNA (siCFL2), and CFL2 protein expression was determined 24 h after transfection by immunoblotting. Intensities were normalized versus β-actin. (**B**) Representative images of FITC-phalloidin (green) and Hoechst 33342 (blue) staining after 24 h of transfection. Scale bar: 25 μm. Phalloidin intensities were analyzed by ImageJ software. (**C**,**D**) YAP and phosphorylated YAP (pYAP) protein expressions in the nuclear and cytoplasmic fractions were determined by immunoblotting after 24 h of transfection with scRNA or miR-325-3p mimic into C2C12 myoblasts. The quality of subcellular fractionation was confirmed using cytoplasmic (α-Tubulin) or nuclear (YY1) markers. Immunoblot results are shown as relative ratios versus scRNA control. All results are presented as the means ± SEMs (*n* > 3), and levels of significance are presented as **, *p* < 0.01; ***, *p* < 0.001 vs. scRNA controls.

In a previous study, knockdown of CFL2 provoked the accumulation of F-actin in myoblasts [25], and thus, we hypothesized that miR-325-3p increases F-actin by inhibiting CFL2 expression in myoblasts. Transfection of myoblasts with siCFL2 significantly decreased CFL2 level by ~60% (Figure 3A) and transfection with miR-325-3p mimic efficiently elevated (>200-fold) the cellular level of miR-325-3p in myoblasts (data not shown). Under this experimental condition, miR-325-3p mimic or siCFL2 dramatically increased F-actin as determined with FITC-coupled phalloidin (Figure 3B). Because actin levels remained constant during differentiation regardless of treatments, the induction of F-actin accumulation by miR-325-3p mimic was ascribed to lack of actin depolymerization due to CFL2 suppression. Recently, it was reported that F-actin accumulation inhibits phosphorylation of a transcriptional coactivator YAP and induces the nuclear translocation of YAP, leading to activation of proliferative transcriptional programs in the Hippo signaling pathway [31,32]. In the present study, transfection with miR-325-3p mimic decreased the phosphorylation of YAP (pYAP) in the cytosol and redistributed YAP to the nucleus from the cytosol (Figure 3C,D), implying that the effects of miR-325-3p on F-actin and YAP might stimulate the proliferation of C2C12 myoblasts.

### 3.4. MiR-325-3p Promoted Myoblast Proliferation

**Figure 4:**
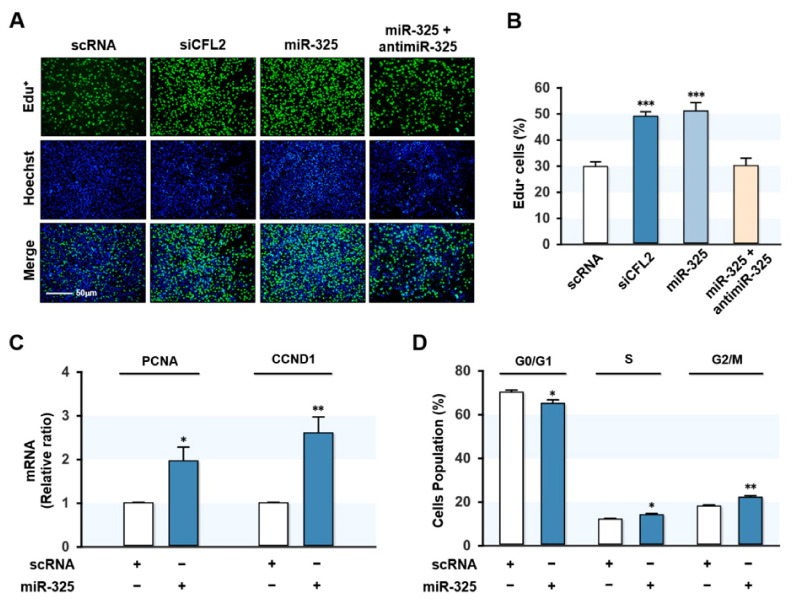
MiR-325-3p promoted myoblast proliferation and cell cycle progression. C2C12 cells were transfected with 200 nM of scRNA, siCFL2, miR-325-3p mimic (miR-325), or antimiR-325-3p (antimiR-325). (**A**) EdU assays were conducted 24 h after transfection. Cells undergoing DNA replication were labeled with EdU (green) and nuclei with Hoechst 33342 (blue). Scale bar: 50 μm. (**B**) Percentages of EdU-positive cells were determined by the ImageJ program. (**C**) PCNA and CCND1 levels in C2C12 myoblasts were determined by *q*RT-PCR and normalized with U6. (**D**) Flow cytometry after 24 h of transfection with scRNA or miR-325-3p mimic. All results are presented as the means ± SEMs (*n* > 3), and levels of significance are presented as *, *p* < 0.05; **, *p* < 0.01; ***, *p* < 0.001 vs. scRNA controls.

To analyze the effect of miR-325-3p on myoblast proliferation, we determined the EdU incorporation in myoblasts after 24 h of siCFL2 or miR-325-3p mimic transfection. Consistent with our previous finding, knockdown of CFL2 by siCFL2 dramatically increased the percentage of EdU-positive cells as compared with scRNA control (Figure 4A,B), indicating increased myoblast proliferation. Interestingly, transfection with miR-325-3p mimic also significantly increased the percentage of EdU-positive myoblasts, whereas co-transfection with antimiR-325-3p completely abolished the increased EdU incorporation by miR-325-3p mimic in myoblasts (Figure 4A,B), suggesting that miR-325-3p mimic promoted myoblast proliferation. Next, we investigated the expressions of genes related to cell proliferation and cell cycle progression, such as proliferating cell nuclear antigen (PCNA) and CCND1, by *q*RT-PCR. Expectedly, PCNA and CCND1 were significantly upregulated in myoblasts transfected with miR-325-3p mimic (Figure 4C). In addition, the effect of miR-325-3p on the cell cycle was assessed by fluorescence-activated cell sorting. As shown in Figure 4D, miR-325-3p mimic transfection lowered the proportion of cells in the G0/G1 phase while increased in the S and G2/M phase, suggesting that miR-325-3p stimulated cell cycle progression. Collectively, miR-325-3p mimic promoted myoblast proliferation and cell cycle progression.

### 3.5. MiR-325-3p Suppressed the Expressions of Myogenic Factors

**Figure 5:**
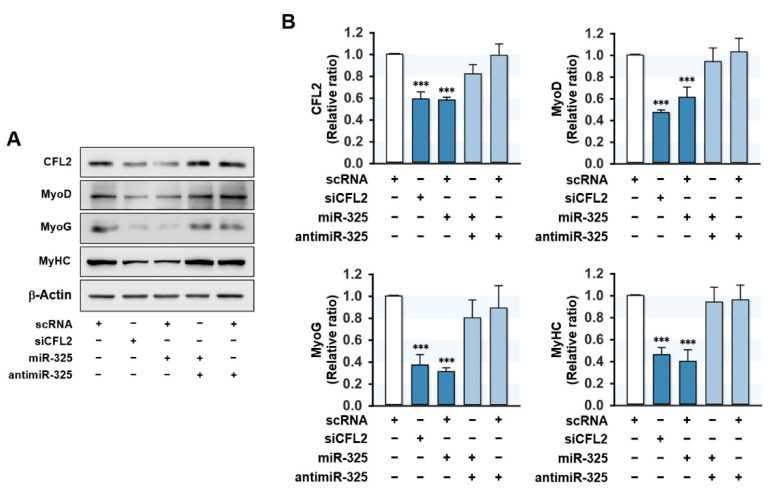
MiR-325-3p suppressed myogenic factors expressions. C2C12 cells were transfected with 200 nM of scRNA, siCFL2, miR-325-3p mimic (miR-325), or antimiR-325-3p (antimiR-325) and differentiated for three days. (**A**) Representative immunoblots of CFL2, MyoD, MyoG, and MyHC levels. (**B**) Quantitative analysis of immunoblots with antibodies for myogenic factors (MyoD, MyoG, and MyHC) and CFL2. Intensities were normalized versus β-actin. All results are presented as the means ± SEMs (*n* > 3), and levels of significance are presented as ***, *p* < 0.001 vs. scRNA controls.

Cell cycle exit and proliferation arrest have long been recognized as necessary conditions for progenitor cell differentiation [2,33]. Therefore, we investigated whether miR-325-3p influences the expressions of myogenic factors. C2C12 cells were transfected with scRNA, siCFL2, miR-325-3p mimic, or antimiR-320a-3p, and the protein expressions of MyoD, MyoG, and MyHC were assessed on differentiation day 3 (Figure 5A,B). As was expected, siCFL2 reduced the level of CFL2 by ~50% versus the scRNA control and significantly reduced the expressions of the myogenic factors MyoD, MyoG, and MyHC. Interestingly, transfection with miR-325-3p mimic also suppressed CFL2 level markedly and reduced the expressions of MyoD, MyoG, and MyHC versus scRNA controls, which suggested that CFL2 inhibition or miR-325-3p mimic are causally linked to the suppressions of myogenic factors. Furthermore, co-transfection with antimiR-325-3p almost completely abrogated the inhibitory effect of miR-325-3p mimic on the expressions of myogenic factors (Figure 5A,B). These results demonstrated that miR-325-3p suppresses myogenic factors expressions in C2C12 myoblasts.

### 3.6. MiR-325-3p Impeded Myogenic Differentiation

**Figure 6:**
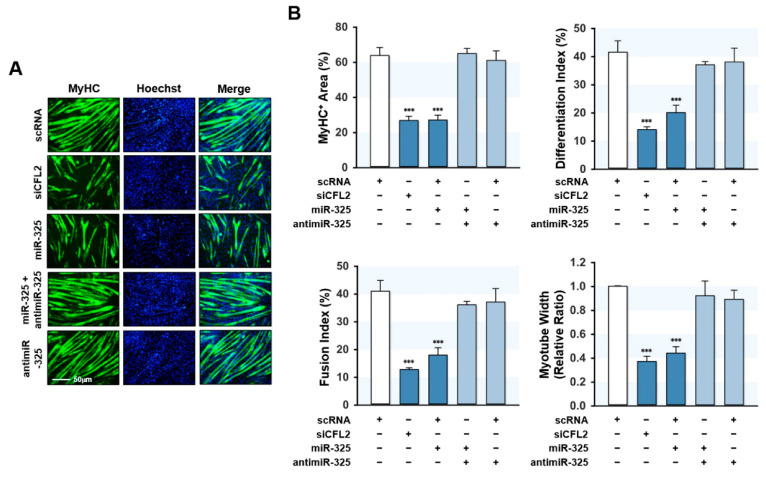
MiR-325-3p negatively regulated myogenic differentiation and myotube formation. C2C12 cells were transfected with 200 nM of scRNA, siCFL2, miR-325-3p mimic (miR-325), or antimiR-325-3p (antimiR-325) and differentiated for five days. (**A**) Representative immunocytochemistry with MyHC antibody (green) and Hoechst 33342 (blue). Scale bar: 50 μm. (**B**) Quantitative analysis of MyHC-positive area, differentiation index, fusion index, and myotube width. All results are presented as the means ± SEMs (*n* > 3), and levels of significance are presented as ***, *p* < 0.001 vs. scRNA controls.

The suppressive effect of miR-325-3p mimic on myogenic factors suggests miR-325-3p might impair myoblast differentiation and myotube formation. Therefore, we transfected C2C12 cells with scRNA, siCFL2, miR-325-3p mimic, or antimiR-325-3p and allowed them to differentiate for five days (Figure 6). Immunocytochemistry with an MyHC antibody was used to analyze myoblast differentiation, followed by quantitative image analysis. CFL2 knockdown by siCFL2 significantly reduced myogenic differentiation, fusion, and myotube formation in myoblasts as determined by differentiation index, fusion index, percentage areas of MyHC-positive cells, and myotube widths, suggesting that CFL2 depletion impaired myoblast differentiation (Figure 6A,B). Similarly, transfection with miR-325-3p mimic also significantly decreased myoblast differentiation and myotube formation as assessed by immunocytochemistry and image analysis (Figure 6A,B). Additionally, co-transfection with antimiR-325-3p abolished the inhibitory effect of miR-325-3p mimic on myoblast differentiation (Figure 6A,B). Thus, these results suggest that miR-325-3p impaired myogenic differentiation and myotube formation in C2C12 myoblasts.

## 4. Discussion

Although miRNAs are known to play a significant role in myogenesis and to be involved in the pathogenesis of muscle wasting [9,10,16], the molecular mechanisms whereby SFA or obesity-induced miRNAs affect myogenesis remain poorly understood. Here, we unveiled an essential role of miR-325-3p on CFL2 expression, myoblast proliferation, and myogenic differentiation, which contributes to our current understanding of the miRNA-mediated myogenic regulatory mechanism in the background of obesity. We summarize our key findings as follows: (i) PA impaired the differentiation of myoblasts, downregulated CFL2, and upregulated miR-325-3p expression; (ii) MiR-325-3p directly targeted the 3′UTR of *CFL2* and suppressed CFL2 expression; (iii) transfection of C2C12 myoblasts with miR-325-3p mimic promoted actin polymerization and induced the nuclear accumulation of YAP; (iv) MiR-325-3p mimic also enhanced myoblast proliferation and cell cycle progression; (v) MiR-325-3p mimic reduced the expressions of myogenic factors and inhibited myogenic differentiation. Therefore, these results indicate that miR-325-3p plays a vital role in myogenesis by regulating the expression of CFL2 and suggest miRNA-mediated myogenic regulation in association with SFA and obesity.

Our results in the current study demonstrate the regulation of myogenesis by miR-325-3p and support our hypothesis that certain miRNAs induced by SFA impair myogenesis. Notably, miR-325-3p markedly upregulated by PA promoted myoblast proliferation and cell cycle progression. Since it has been known that myoblast proliferation and myogenic differentiation are inversely related during myogenesis, proliferation arrest is a prerequisite for the differentiation of myoblasts [2,33]. In this regard, the inhibition of myogenic differentiation by miR-325-3p is primarily attributed to the promotion of cell cycle progression and proliferation in myoblasts. Interestingly, the upregulation of miR-325-3p has been implicated in the occurrence and progression of various malignancies [34,35,36,37], and miR-325-3p overexpression promoted cancer cell proliferation, invasion, and metastasis [34]. Although several other studies showed the suppressive effect on proliferation by miR-325-3p in cancer cells [38,39,40], this discrepancy regarding the effect of miR-325-3p on cell proliferation might be explained by the cell type-dependent differences in composition of protein components, target proteins abundance, and miR-325-3p level. In this respect, it is worth noting that CFL2 as a target of miR-325-3p is a skeletal muscle-specific protein that is upregulated in myoblasts during myogenic differentiation [19,25].

Then, what mechanism is responsible for miR-325-3p-induced myoblast proliferation and cell cycle progression? According to one of the important findings of the present study, miR-325-3p promoted F-actin formation by directly inhibiting the expression of CFL2 (Figure 3). CFL2 has been recognized as a necessary component of actin remodeling due to its ability to sever F-actin, which regulates mechanical stress in the cytoskeleton [20,24]. The actin cytoskeleton dynamics has been suggested to be a critical regulator of YAP in the Hippo signaling pathway [41], which controls tissue and organ sizes in animals by modulating cell proliferation and differentiation [42]. The nuclear translocations of cytosolic YAP and TAZ activate proliferative and anti-apoptotic transcriptional activities in this pathway [43]. Furthermore, F-actin accumulation was shown to diminish the phosphorylation of YAP/TAZ and, consequently, increases their nuclear translocation and cell proliferation [31,32]. In this regard, F-actin severing proteins such as CFL and Gelosin act as negative regulators of YAP by increasing its phosphorylation and degradation [23,44]. Accordingly, actin remodeling mediated by CFL is directly connected to the regulation of cell proliferation via the nuclear translocation of YAP [23,24]. In a previous study, we found knockdown of CFL2 resulted in F-actin accumulation and increased cell cycle progression and cell proliferation in C2C12 myoblasts [25]. Torrini et al. also discovered that CFL2 depletion enhanced F-actin levels and activated YAP in cardiomyocytes [45]. Moreover, cytochalasin D, a potent actin depolymerizer, inhibited the nuclear translocation of YAP, whereas jasplakinolide, an F-actin inducer, promoted its nuclear translocation [45]. Our data suggest that the stimulatory effect of miR-325-3p on cell proliferation is primarily related to the disruption of actin dynamics caused by CFL2 suppression. Collectively, miR-325-3p inhibited CFL2 expression, elevated F-actin accumulation, induced the nuclear translocation of YAP, and ultimately led to myoblast proliferation and delayed myogenic differentiation.

Although the regulatory mechanism responsible for miR-325-3p induction by PA was not investigated in this work, we speculate that specific transcription factors activated by PA or obesity may mediate the upregulation of miR-325-3p in myoblasts. To address this issue, we analyzed the promoter regions of human and mouse miR-325-3p and found an optimal consensus binding site for the E2F1 transcription factor. E2F1, a member of the E2F family of transcription factors, has often been implicated in metabolic regulation and acts as a pivotal player in the cell cycle progression for cell growth and survival [46]. Previously, Bo et al. showed E2F1 bound to miR-325-3p promoter and enhanced miR-325-3p expression in cardiomyocytes, and E2F1 knockout mice exhibited a low miR-325-3p level, indicating that E2F1 is a transcriptional activator of miR-325-3p [47]. Interestingly, E2F1 levels were elevated in the adipose tissue of obese humans [48] and obese mouse models, such as high-fat diet (HFD)-fed mice and ob/ob mice [49]. Given the functions and regulation of E2F1 in proliferation and metabolism, it appears that E2F1 might play a critical role in the upregulation of miR-325-3p in obesity. Another interesting recent study demonstrated that cellular treatment of transforming growth factor-β (TGF-β) increased miR-325-3p expression in colorectal carcinoma cells [35]. TGF-β is a well-known key modulator of insulin resistance in metabolic disorders associated with obesity [50]. Indeed, circulating TGF-β levels were increased in obese humans, ob/ob mice, and HFD-induced obese mice [51]. Although further study is warranted, the results of previous studies suggest that the activation of E2F1 or TGF-β in a background of obesity may induce miR-325-3p expression, thereby provoking impaired myogenesis and muscle wasting.

## 5. Conclusions

This study demonstrates that miR-325-3p plays an essential role in actin remodeling and myogenic differentiation in C2C12 myoblasts. PA inhibited differentiation of myoblasts and induced miR-325-3p expression. Interestingly, miR-325-3p inhibited the expression of CFL2, which is required for myogenic differentiation, via directly targeting the 3′UTR of *CFL2* mRNA. Transfection of miR-325-3p mimic increased F-actin and stimulated the nuclear translocation of YAP, thus promoting myoblast proliferation and impaired myogenic differentiation. The roles of miR-325-3p on CFL2 expression and myogenic differentiation suggest a novel miRNA-mediated mechanism that regulates myogenesis in the background of obesity. From a clinical point of view, miR-325-3p may be a vital mediator between obesity and muscle wasting and will provide a means of developing practical diagnostic and therapeutic approaches for muscle wasting and sarcopenic obesity.

## Supplementary Materials

The following are available online at https://www.mdpi.com/article/10.3390/cells10102725/s1, Supplementary data: Analysis of miR-325-3p expression using an Affymetrix GeneChip miRNA 4.0 array, Table S1: Oligonucleotide sequences for transfection, Table S2: Primer lists and PCR conditions for qRT-PCR, RT-PCR and cloning, Table S3: Antibodies list.
