# Supplementary material for: Role of MiR-325-3p in the Regulation of CFL2 and Myogenic Differentiation of C2C12 Myoblasts
Source: Cells. 2021 Oct 12;10(10):2725. doi: 10.3390/cells10102725 (PMC8534702; doi:10.3390/cells10102725)
Supplement: Supplementary file 1 [file cells-10-02725-s001.zip › cells-1365395-supplementary.pdf]

cells

MDPI

Article

Role of MiR-325-3p in the Regulation of CFL2 and Myogenic Differentiation of C2C12 Myoblasts

Mai Thi Nguyen [1] and Wan Lee [1,2,*]

[1] Department of Biochemistry, Dongguk University College of Medicine, 123 Dongdae-ro, Gyeongju 38066, Korea; nguyenmainhp@gmail.com
[2] Channelopathy Research Center, Dongguk University College of Medicine, 32 Dongguk-ro, Ilsan Dong-gu, Goyang 10326, Korea
* Correspondence: wanlee@dongguk.ac.kr; Tel.: +82-54-770-2409

Supplementary Data: Analysis of miR-325-3p expression using an Affymetrix GeneChip miRNA 4.0 array

1. PA treatment, RNA extraction, and quality check

A fatty acid-free bovine serum albumin (BSA, Bovogen, VIC, Australia)-conjugated PA (Sigma-Aldrich) solution was prepared. Briefly, BSA and sodium PA were entirely dissolved in 150 mM NaCl by heating at 37°C and 70°C, respectively. The BSA solution was added dropwise to the PA solution at 37°C with continuous stirring until the PA to BSA molar ratio was 6:1. The BSA-conjugated PA and BSA vehicles were aliquoted and stored at -80°C. C2C12 cells were seeded on 6-well plates (Thermo Fisher Scientific) at a density of 1.3×10^5 cells/well in 2 ml of GM. After 24 h, they were treated with BSA-conjugated PA (100 μM) for 24 h as described previously [1]. The control cells were treated with the BSA vehicle. The total RNA from the C2C12 cells was extracted using a miRNeasy Mini Kit (Qiagen) according to the manufacturer's instructions. The purity and integrity of the RNA were assessed using an ND-1000 Spectrophotometer (NanoDrop) and Agilent 2100 Bioanalyzer (Agilent Technologies). Equal amounts of RNA from three independent experiments were pooled together and used for the microarray.

2. miRNA arrays analysis

The total RNA described above was prepared and subjected to an Affymetrix GeneChip miRNA 4.0 array (Affymetrix, Santa Clara, CA, USA) process according to the Affymetrix technical instructions. Briefly, 600ng RNA was labeled with a FlashTag™ Biotin RNA Labeling Kit (Genisphere, Hatfield, PA, USA). The labeled RNA was quantified, fractionated, and hybridized to the miRNA microarray according to the manufacturer's instructions. The labeled RNA was heated to 99°C for 5 min and incubated at 45°C for 5 min. RNA-array hybridization was conducted with agitation at 60 rpm for 16 hrs at 48°C on an Affymetrix® 450 Fluidics Station. The chips were stained on a GeneChip Fluidics Station 450 (Affymetrix) and scanned using an Affymetrix GCS 3000 scanner (Affymetrix).

According to the quantile method after a log 2 transformation, all signals were normalized to make them comparable across microarrays.

3. Expression level of miR-325-3p.

ProbeID	Transcript	**Palmitate_0.1 mM/Palmitate_0 mM. fold change**	Palmitate_0.1mM/Palmitate_0mM. volume	N_Palmitate_0mM	N_Palmitate_0.1mM	160712_1_Palmitate_0mM_(miRNA-4_0).rma-dabg-Signal	160712_2_Palmitate_0.1mM_(miRNA-4_0).rma-dabg-Signal	160712_1_Palmitate_0mM_(miRNA-4_0).rma-dabg-Detection	160712_2_Palmitate_0.1mM_(miRNA-4_0).rma-dabg-Detection	fail.count	GeneChip Array
20501311	miR-325	**2.20105**	1.4429149	0.9819918	2.120184	0.9819918	2.120184	P	P	0	miRNA-4_0

Reference

[1] M.T. Nguyen, K.H. Min, W. Lee, MiR-96-5p Induced by Palmitic Acid Suppresses the Myogenic Differentiation of C2C12 Myoblasts by Targeting FHL1, Int J Mol Sci, 21 (2020).

Table S1. Oligonucleotide sequences for transfection

Gene	Primer sequence (5′-3′)
scRNA (control RNA)	UCACAACCUCCUAGAAAGAGUAGA
siCFL2	GCUCUAAAGAUGCCAUUAAUU
miR-325-3p	AAAAGCUGGGUUGAGAGGGCGA
antimiR-325	Genolution

Table S2. Primer lists and conditions for *q*RT-PCR, RT-PCR and cloning

(A) Mouse primer lists for *q*RT-PCR and RT-PCR

Gene	Primer sequence (5′-3′)		Product size	Annealing Temperature	Concentration		Cycle
					cDNA	Primer	
miR-325-3p	F.P	CCTAGTAGGTGTCCAGTAAGTGT	90	55	2 ng/μl	0.5 μM	40
miRNA universal Primer	R.P	miScript universal primer (Qiagen)					
U6	F.P	CTCGCTTCGGCAGCACA	94				
	R.P	AACGCTTCACGAATTTGCGT					
β-Actin	F.P	TCACCCACACTGTGCCCATCTACGA	348	58			
	R.P	GGATGCCACAGGATTCCATACCCA					
CFL2	F.P	CCGACCCCTCCTTCTTCTCG	100	58			
	R.P	GTAACTCCAGATGCCATAGTG					
CCND1	F.P	ACCAATCTCCTCAACGACCG	228	58			
	R.P	ACGGAAGGGAAGAGAAGGG					
PCNA	F.P	GAACCTGCAGAGCATGGACTC	201	58			
	R.P	GGTGTCTGCATTATCTTCAGCCC					

(B) Primer lists for wild-type and mutant 3'UTR cloning

Gene	Primer sequence (5′-3′)		Product size	Annealing Temperature	Concentration		Cycle
					cDNA	Primer	
CFL2*wt*	F.P	CATTCCTGTTACCTGCATATCTTCT	358	58	2 ng/μl	0.5 μM	35
	R.P	TCTCTGCACTGGTCATTTGA					
CFL2*mut*	F.P	CGTTGCAGATAAAATTGTGGCAT	128				
	R.P	TCTCTGCACTGGTCATTTGA					
	F.P	CATTCCTGTTACCTGCATATCTTCT	253				
	R.P	ATGCCACAATTTTATCTGCAACG					

Table S3. Antibodies list

Antibody	Type	Targeted species	Manufacturer	Cat. No.	Dilution ratio*
CFL2	Polyclonal	Rabbit	Lifespan Biosciences, Seatlle, WT, USA	LS-C409553	1:2,000
MyHC	Monoclonal	Mouse	DSHB, Iowa, IA, USA	MF20	1:1,000
MyoD	Monoclonal	Mouse	Santa Cruz Biotechnology, Dallas, TX, USA	sc-377460	1:1,000
MyoG	Monoclonal	Mouse	Santa Cruz Biotechnology, Dallas, TX, USA	sc-12732	1:1,000
YAP	Monoclonal	Rabbit	Cell Signaling Technology, Danvers, MA, USA	14074S	1:10,000
pYAP	Polyclonal	Rabbit	Cell Signaling Technology, Danvers, MA, USA	4911S	1:10,000
YY1	Polyclonal	Rabbit	Santa Cruz Biotechnology, Dallas, TX, USA	sc-1703	1:5,000
α-Tubulin	Monoclonal	Mouse	DSHB, Iowa, IA, USA	12G10	1:2,000
β-actin	Monoclonal	Rabbit	Sigma-Aldrich Chemical, St. Louis, USA	A2066	1:10,000
Antibodies HRP-linked anti-rabbit IgG			Cell Signaling Technology, Danvers, MA, USA	#7074	1:10,000
Goat anti-mouse(H+L)			Invitrogen, Thermofisher Scientific, Waltham, MA USA	#32430	1:2,000

*All blots were visualized using a Femto reagent (Thermofisher Scientific).
